# A Novel Role of Human Holliday Junction Resolvase GEN1 in the Maintenance of Centrosome Integrity

**DOI:** 10.1371/journal.pone.0049687

**Published:** 2012-11-16

**Authors:** Min Gao, Jannie Rendtlew Danielsen, Lei-Zhen Wei, Dong-Ping Zhou, Qian Xu, Miao-Miao Li, Zhao-Qi Wang, Wei-Min Tong, Yun-Gui Yang

**Affiliations:** 1 Disease Genomics and Individualized Medicine Laboratory, Beijing Institute of Genomics, Chinese Academy of Sciences, Beijing, P. R. China; 2 The Novo Nordisk Foundation Center for Protein Research, University of Copenhagen, Copenhagen, Denmark; 3 Leibniz Institute for Age Research-Fritz Lipmann Institute e.V. and Faculty of Biology-Pharmacy, Friedrich-Schiller-University, Jena, Germany; 4 Department of Pathology and Center for Experimental Animal Research, Chinese Academy of Medical Sciences and Peking Union Medical College, Beijing, P. R. China; 5 University of Chinese Academy of Sciences, Beijing, P. R. China; Institut de Génétique et Développement de Rennes, France

## Abstract

The maintenance of genomic stability requires accurate genome replication, repair of DNA damage, and the precise segregation of chromosomes in mitosis. GEN1 possesses Holliday junction resolvase activity *in vitro* and presumably functions in homology driven repair of DNA double strand breaks. However, little is currently known about the cellular functions of human GEN1. In the present study we demonstrate that GEN1 is a novel centrosome associated protein and we characterize the various phenotypes associated with GEN1 deficiency. We identify an N-terminal centrosome localization signal in GEN1, which is required and sufficient for centrosome localization. We report that GEN1 depletion results in aberrant centrosome numbers associated with the formation of multiple spindle poles in mitosis, an increased number of cells with multi-nuclei, increased apoptosis and an elevated level of spontaneous DNA damage. We find homologous recombination severely impaired in GEN1 deficient cells, suggesting that GEN1 functions as a Holliday junction resolvase *in vivo* as well as *in vitro*. Complementation of GEN1 depleted cells with various GEN1 constructs revealed that centrosome association but not catalytic activity of GEN1 is required for preventing centrosome hyper-amplification, formation of multiple mitotic spindles, and multi-nucleation. Our findings provide novel insight into the biological functions of GEN1 by uncovering an important role of GEN1 in the regulation of centrosome integrity.

## Introduction

DNA double strand breaks (DSBs) represent some of the most cytotoxic lesions and pose a major threat to genome stability if not properly repaired [1]–[3]. DSBs are primarily repaired by two mechanisms: non-homologous end joining and homologous recombination (HR) [4], [5]. Non-homologous end joining pieces the DNA ends together in an efficient but error prone fashion. In contrast, HR is an error free way of repair but it requires a sister chromatid as template and is therefore restricted to the S/G2 phase of the cell cycle. Following recognition and initial resection of the DSB by the MRN (Mre11–Rad50–NBS1) complex, broken ends are further resected by BLM and EXO1 to generate 3′ single-strand overhangs [6]–[8]. RPA molecules initially coat these tails, but become replaced by Rad51 forming a nucleofilament on the 3′ ssDNA overhang, which together with Rad52/Rad54/BRCA2 promote homologous strand invasion to form a D-loop structure [9]. Migration of the D-loop results in capture of the second end of the DSB and subsequent ligation leads to the formation of the double Holliday junction (dHJ) structure. The dHJ can be resolved either through cleavage by HJ resolving enzymes or through ‘‘dissolution’’ by the BLM-TopIII complex [10]–[14]. Classical HJ resolving enzymes are nucleases that resolve dHJs by introducing two perfectly symmetrical cleavages that result in either crossover or non-crossover products, depending on the orientation of the cleavage site. Currently two enzymes with classical HJ resolvase activity have been identified: the SLX4 scaffold protein in association with the SLX1 nuclease and GEN1 [15]–[17]. In addition, the conserved Mus81-Eme1 complex can process HJ intermediates such as nicked HJs [14], [18], [19]. Though, the biochemical function of human GEN1 has been very well characterized *in vitro* the cellular functions of human GEN1 are less clear [17], [20], [21]. However, a recent study showed that co-depletion of GEN1 and Mus81 or SLX4 in Bloom’s syndrome cells resulted in severe defects in chromosome condensation, presumably due to unresolved Holliday junctions, when all three Holliday junction dissolution/resolution pathways were compromised [22]. In addition, ectopic expression of human GEN1 in fission yeast, which does not encode a *gen-1/yen1* homolog, complements the meiotic defect observed in mus81 mutants, suggesting that GEN1 can function in HJ resolution *in vivo* [23]. Surprisingly, deletion of *yen1* in budding yeast resulted in no obvious DNA repair defect, but when combined with deletion of Mus81 an increased sensitivity to MMS was observed [21]. A recent study in *C. elegans* indicated that defects in IR-induced cell cycle arrest and apoptosis in *gen-1* mutants were explained not only by the role of GEN1 in HR but also by GEN1 mediated DNA damage signaling suggesting a broader role of GEN1 in the maintenance of genomic stability [24].

Defective DNA repair including repair defects caused by depletion of DSB repair proteins such as ATR, BRCA1/2, Rad54, Rad51 and its homologs has been shown to result in centrosome amplification [25]–[32]. This indicates that centrosome copy number control and hence normal chromosome segregation may be coupled to the function of repair proteins. The centrosome constitutes the primary microtubule organizing center in the cell. It is composed of two barrel-shaped centrioles embedded within pericentriolar material. Correct duplication of the centriole during S-phase and the formation of the bipolar mitotic spindle are important for appropriate segregation of the chromosomes during cell division [33]. Centrosome amplification can occur in several ways. Numerical centrosome aberrations can arise after failed cytokinesis [34]. Extended G1/S arrest can decouple the centrosome and chromosome cycles [26], [35]–[37]. The frequent centrosome abnormalities seen in p53-deficient cells [38], in cells with DNA repair deficiencies [25]–[27], in cells after DNA damage induction [31], and in cells with telomere defects [39] have been suggested to be caused by duplication in G2/M [26], [31].

Here, we demonstrate that GEN1 is a novel centrosome associated protein. We show that GEN1 depletion leads to an increase in centrosome numbers, resulting in the formation of multiple functional mitotic spindles, multi-nucleation and cell death. Furthermore, we find that GEN1 depletion results in a significant reduction in HR as well as increased spontaneous DNA damage providing evidence for a function of human GEN1 in HR *in vivo.* Interestingly, the function of GEN1 in HR appears separable from its function in maintaining centrosome integrity.

## Results

### GEN1 Localizes to the Centrosome

**Figure 1:**
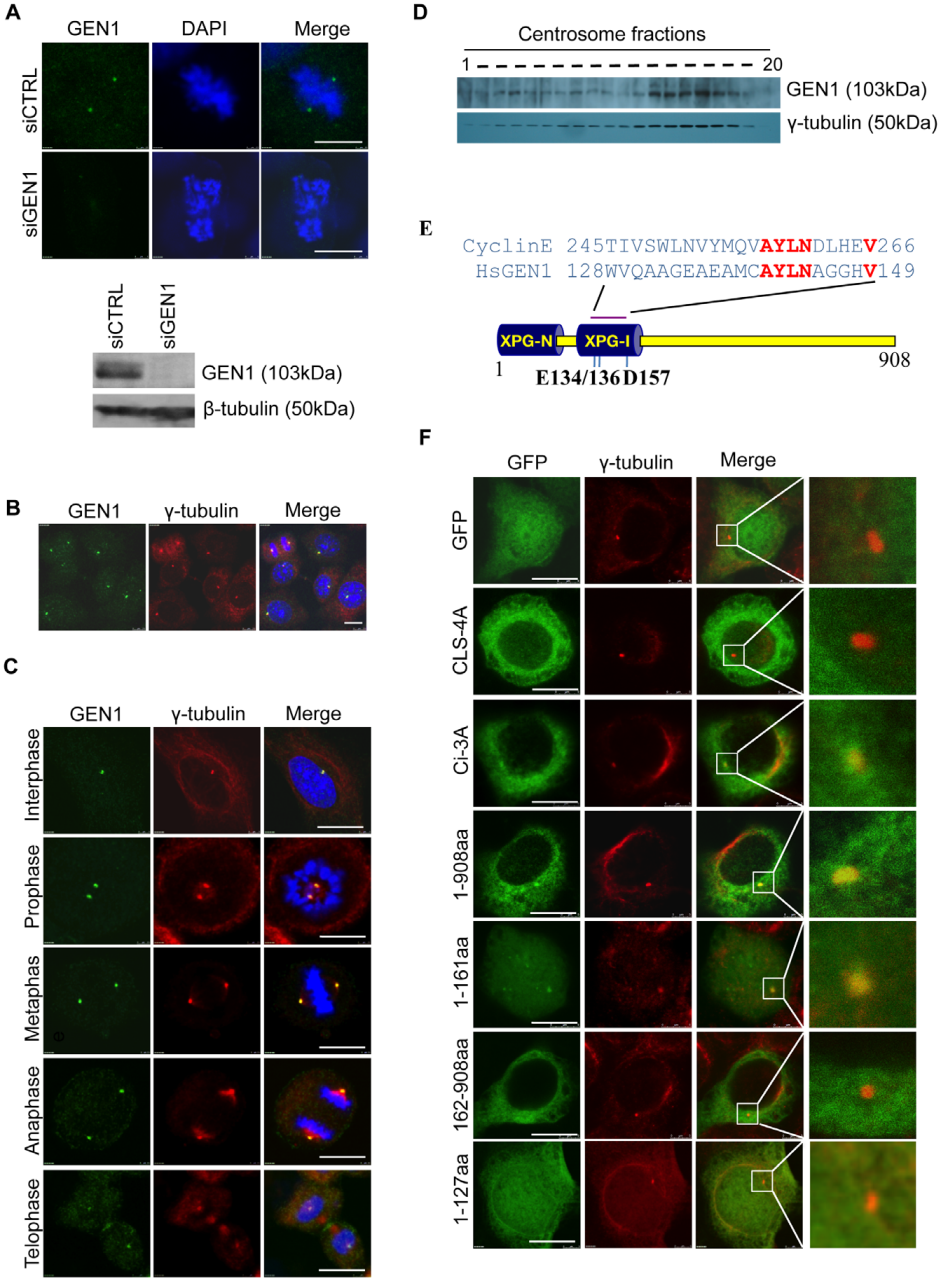
Human GEN1 localizes on the centrosome. A. (top) MRC5 cells were fixed with methanol and immunostained with GEN1 antibody (GST-GEN1 651–892 antibody) and DAPI. Scale bars, 10 µm. (bottom) Cells were lysed and separated by SDS-PAGE and immunoblotted using the indicated antibodies. **B.** HeLa cells were fixed with methanol and immunostained with anti-GEN1 (651–892aa) antibody, anti-γ-tubulin antibody and DAPI. Scale bars, 10 µm. **C.** Hela cells at different phases of the cell cycle were fixed with methanol and co-immunostained with the indicated antibodies and DAPI. Scale bars, 10 µm. **D.** Centrosomes were isolated from HeLa cells and purified fractions from a discontinuous sucrose gradient were separated by SDS-PAGE and immunoblotted using the indicated antibodies. **E.** Schematic showing GEN1 domains and alignment of the Cyclin E CLS and the putative CLS in GEN1. Highly conserved amino acids are shown in red. In black are shown three of the key amino acids within the endonuclease domain of GEN1. XPG (-N: N-terminal, -I, internal) nuclease domain domain very similar to the nuclease domain first identified in XPG. **F.** HeLa cells were transfected with the indicated plasmids and 24 h later methanol fixed and immonostained with γ-tubulin and DAPI. GEN1 was detected by GFP fluorescence. Scale bars, 10 µm. Images to the left are zoomed 27.5×. CLS-4A: centrosome localization defective mutant, Ci-3A: catalytic inactive mutant.

To investigate the role of GEN1 in human cells, we first generated anti-GEN1 polyclonal antibodies (**Fig. S1A–B**). Immunofluorescence microscopy revealed pronounced and specific centrosome-like accumulation of GEN1 (**Fig. 1A**). Co-localization with γ-tubulin confirmed that these GEN1-decorated organelles were indeed centrosomes (**Fig. 1B**). The centrosomal association of GEN1 was seen in several cell lines, with different antibodies (N- and C-terminal peptide generated antibodies **Fig. S1C–D**), and was independent of fixation methods (**Fig. S2A–S2C**). Finally, the localization of GEN1 on the centrosome was unaltered by a 14 h treatment with the microtubule-depolymerizing drug nocodazole (**Fig. S2D**), suggesting that GEN1 interacts with genuine centrosome components and that retention at centrosomes does not depend on microtubule targeting. According to immunostainings of exponentially growing MRC5 cells captured in interphase and at various stages of mitosis, the centrosomal localization of GEN1 is not regulated during the cell cycle (**Fig. 1C**). In addition, sucrose-density-gradient purification of centrosomes from HeLa cells revealed that a pool of GEN1 co-fractionated with the centrosomal protein γ-tubulin consistent with the immunostainings (**Fig. 1D**).

With the intention of identifying the region of GEN1 responsible for its localization to the centrosome we performed sequence alignment of GEN1 with the previous identified centrosome localization signal (CLS) of Cyclin E (**Fig. 1E**) [40]. A putative CLS (128WVQAAGE AEAMCAYLNAGGHV148) containing five of the conserved amino acids found in the Cyclin E CLS was identified in the N-terminus of GEN1, and alignment of various GEN1 orthologs revealed this amino acid cluster to be highly conserved through evolution (**Fig. S2E**). Strikingly, mutation of four of the conserved amino acids (Y141A/L142A/N143A/V148A) completely abrogated GEN1 centrosome accumulation, suggesting that this is indeed a CLS and that it confers the centrosome accumulation ability of GEN1 (**Fig. 1F**). The use of GFP-GEN1 deletion mutants confirmed that the N-terminus of GEN1 is required and sufficient for centrosome targeting (**Fig. 1F**). The nuclease activity of GEN1 was not required for centrosome localization, since catalytic inactive GFP-GEN1 (Ci-3A) accumulated on centrosomes in a manner similar to GFP-GEN1 wild type (WT) (**Fig. 1F**). Mutation of these 3 key amino acids (E134/E136/D157) in the nuclease domain of GEN1 has previously been published to result in a complete disruption of the catalytic activity [17].

### GEN1 Deficiency Leads to Supernumerary Centrosomes in Mitosis

**Figure 2:**
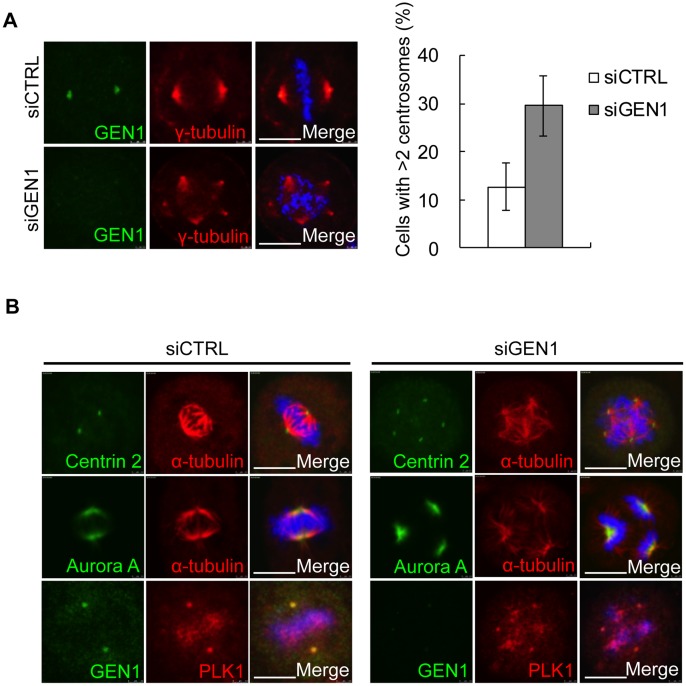
GEN1 deficiency leads to supernumerary centrosomes in mitosis. A. (left) HeLa cells were treated with GEN1 or control (CTRL) siRNA for 48 h, methanol fixed and immunostained with the indicated antibodies. (right) Graph represents the mean of three independent experiments. (n = 300 cells/condition/experiment). Scale bar, 10 µm. **B.** HeLa cells treated with CTRL or GEN1 siRNA were methanol fixed and immunostained with anti-GEN1 (651–892aa) antibody and anti-γ-tubulin antibody. Scale bars, 10 µm.

**Figure 3:**
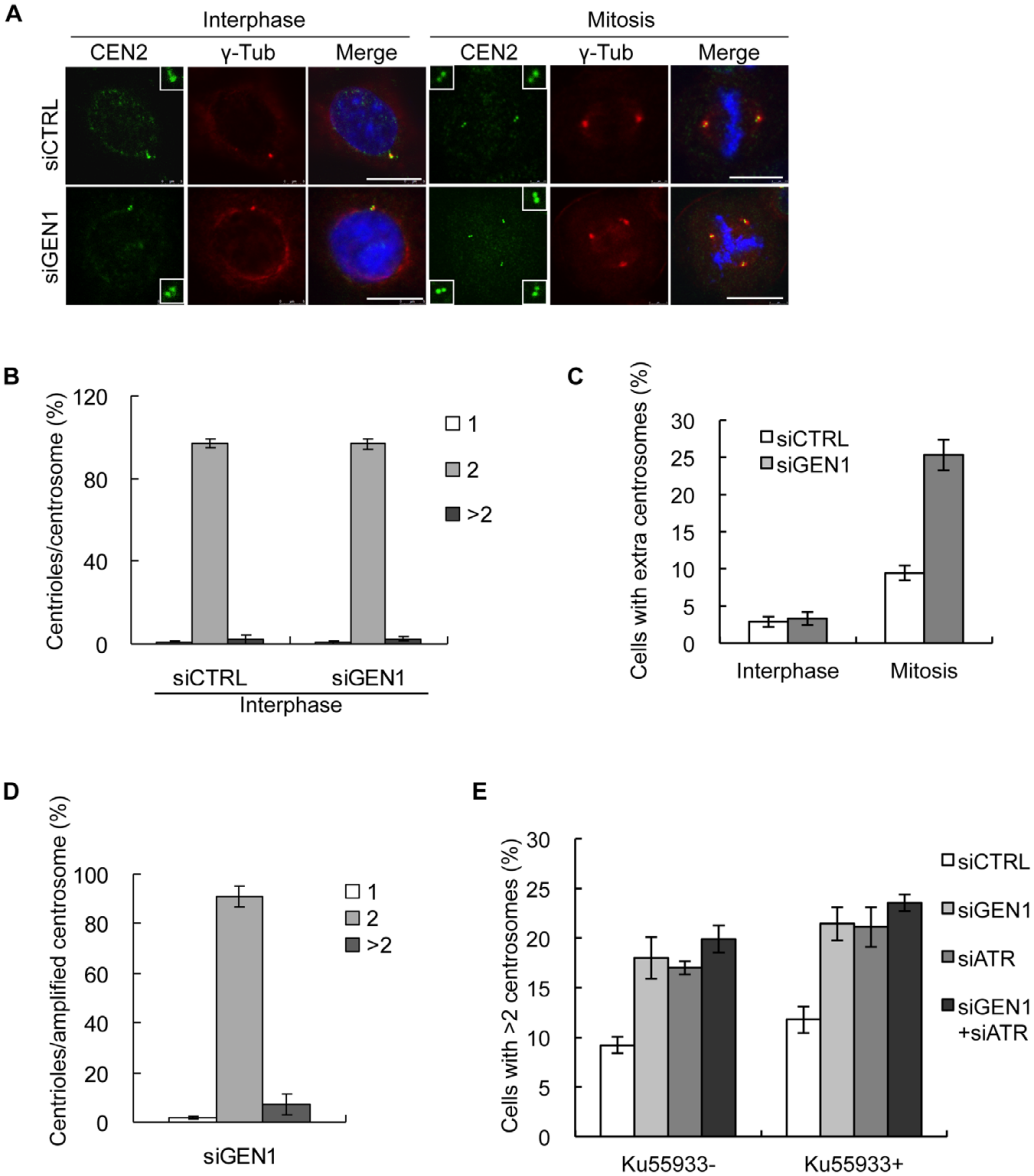
GEN1 depletion results in supernumerary centrosomes in late G2 or mitosis. A. HeLa cells were treated with GEN1 or control (CTRL) siRNA for 48 h, methanol fixed and immunostained with the indicated antibodies. Identification of centrosomes and centrioles was based on immunostaining with γ-tubulin and Centrin 2 antibodies, respectively. Scale bar, 10 µm. **B.** Quantification of centrosomes with 1, 2 or more than 2 centrioles in CTRL or GEN1 depleted interphase cells. Graph represents the mean of three independent experiments. (n = 300 centrosomes/condition/experiment). Error bars indicate S.E.M. **C.** Quantification of interphase and mitotic cells (**Fig. 3A**) with extra centrosomes (interphase>1 centrosome (G1) or>1 linked centrosome pair (S-G2), mitosis>2 separated centrosomes). Graph represents the mean of three independent experiments (n = 300 cells/condition/experiment). Error bars indicate S.E.M. **D.** The percent of supernumerary centrosomes with 1, 2 or more than 2 centrioles after GEN1 depletion. Graph represents the mean of three independent experiments. (n = 300 centrosomes/condition/experiment). Error bars indicate S.E.M. **E.** Quantification of mitotic cells with>2 mitotic spindles 48 h after treatment with CTRL, GEN1 and/or ATR siRNA followed by incubation with or without ATM inhibitor(10 uM Ku55933. Mitotic spindles were visualized by GEN1 (anti-GEN1 (651–892aa) antibody) and γ-tubulin immunostaining. Graph represents the mean of three independent experiments. (n = 300 cells/condition/experiment). Error bars indicate S.E.M.

Having established GEN1 as a novel centrosome associated protein we next examined the impact of GEN1 deficiency on centrosome integrity. 48 h after treatment with GEN1 siRNA, cells were fixed and stained with γ-tubulin and GEN1 antibodies. Interestingly, we observed that GEN1 depletion resulted in an almost 3 fold increase in the number of mitotic cells with multipolar mitotic spindles (>2 mitotic spindles) (**Fig. 2A**). This was repeated and confirmed in several cell lines and with independent siRNAs (**Fig. S3A–C**). SLX4 depleted cells did not exhibit an increase in multipolar spindles demonstrating that the role of GEN1 in maintaining centrosome copy number is not a common function of the HJ resolvases (**Fig. S3D**). Supernumerary centrosomes can arise as a consequence of centrosome fragmentation or splitting, which may yield either functional or non-functional centrosomes. In addition, failed cytokinesis or centrosome over-duplication can also lead to an increased number of centrosomes [26], [35], [37], [41], [42]. To determine if the supernumerary centrosomes were functional we examined the mitotic spindle structure in GEN1 depleted cells. We found that GEN1 deficient cells were as capable as GEN1 proficient cells in nucleating microtubules and generate functional mitotic spindles as determined by α-tubulin immunostaining (**Fig. 2B**). Consistently, normal localization of the centrosome associated proteins Aurora A and Plk1 and the centriolar protein Centrin 2 (**Fig. 2B**), also suggested that centrosome functionality was sustained. To more carefully characterize how GEN1 contributes to the formation of supernumerary centrosomes, we analyzed centrosome integrity in interphase and mitotic cells by combining immunofluorescence staining for Centrin 2 and γ-tubulin (**Fig. 3A–D**). We found that the majority of cells in interphase regardless of whether they had been treated with GEN1 or CTRL siRNA had either one centrosome containing two centrioles (G1) or one linked centrosome pair with two centrioles per centrosome (S-G2) (**Fig. 3B**). Furthermore, the increase in centrosome numbers caused by GEN1 deficiency was only observed in mitotic cells (**Fig. 3C**). These findings implied two things. First, the increase in centrosome numbers in response to GEN1 depletion is most likely not caused by failed cytokinesis, because this would result in interphase cells having more than one centrosome each containing two centrioles. This corresponds well with the fact that we most often observed an unequal number of mitotic spindles in cells with an abnormal mitotic spindle apparatus (Unpublished observations). Second, the formation of supernumerary centrosomes most likely takes place in late G2- or M-phase. However, it was still not clear if the amplified centrosomes had arisen by centrosome fragmentation or duplication. To address this, we carefully counted the number of centrioles in each amplified centrosome following GEN1 siRNA treatment (**Fig. 3D**). We found that more than 90% of the centrosomes found in mitotic cells with supernumerary centrosomes contained 2 centrioles. Around 7% of the centrosomes had more than 2 centrioles while less than 2% was observed to contain one centriole. We did not observe centrosomes without centrioles. Taken together, these findings strongly suggest that the increase in centrosome numbers in response to GEN1 depletion is caused mainly by centrosome duplication during late G2 phase or early mitosis. Several studies have shown that centrosome amplification can be caused by induced DNA damage or by increased spontaneous DNA damage, and that such amplifications are dependent on an ATR/ATM mediated G2/M checkpoint [26], [31], [43]. However, this does not appear to be the case for amplifications caused by GEN1 depletion, since neither ATM inhibitor, ATR depletion nor a combination of these treatments could alleviate the formation of supernumerary centrosomes in GEN1 knockdown cells (**Fig. 3E** and **Fig. S4A**).

### Increased Multi-nucleation and Apoptosis in GEN1 Deficient Cells

**Figure 4:**
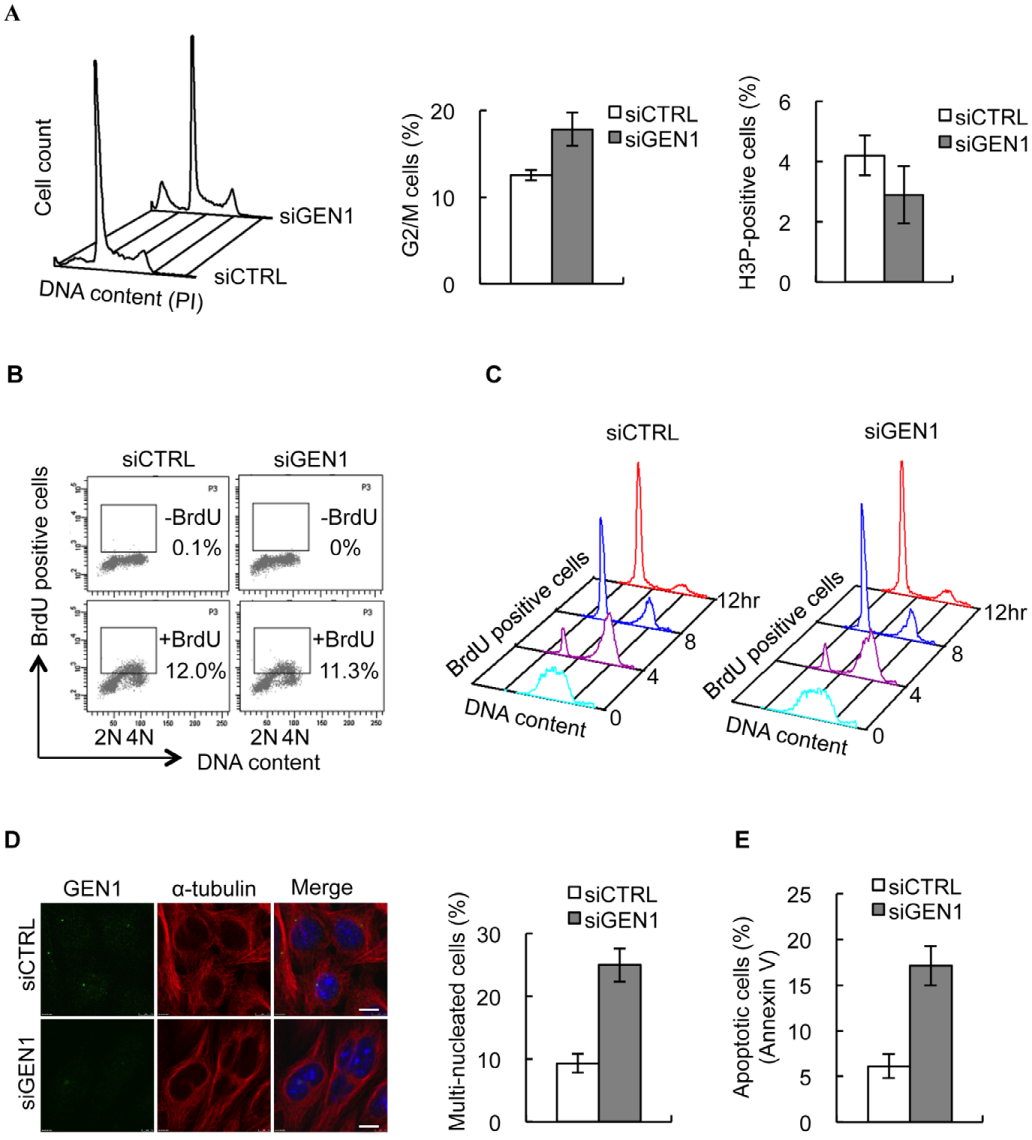
GEN1 depletion interferes with cell cycle progression and results in multi-nucleation and apoptosis. A. (left) 293T cells were treated with CTRL or GEN1 siRNA for 48 h stained with propidium iodine (PI) and analyzed by flow cytometry. (middle) G2/M cells were identified using Modfit software. (right) Mitotic cells were detected by phospho H3 antibody staining followed by flow cytometric analysis. Quantification is based on three independent experiments. Error bars indicate S.E.M. **B.** Hela cells were treated with CTRL or GEN1 siRNA for 48 h, labeled with or without 10 uM BrdU for 2 hours, stained with FITC conjugated anti-BrdU antibodies and propidium iodine (PI), and analyzed by flow cytometry. x-axis: propidium iodine (PI). y-axis FITC. **C.** CTRL or GEN1 siRNA treated Hela cells were incubated with 10 uM BrdU for 2 hours, and return to BrdU-free medium for the indicated times. Cell cycle progression of BrdU labeled cells were analyzed by flow cytometry. **D.** HeLa cells treated with CTRL or GEN1 siRNA (48 h) were methanol fixed and immunostained with GEN1(anti-GEN1 (651–892aa) antibody) and α-tubulin. DNA was visualized by DAPI. (Right) Graph represents the mean of three independent experiments. (n = 100 cells/condition/experiment). Error bars indicate S.E.M. **E.** HeLa cells were treated with CTRL or GEN1 siRNA for 48 h fixed, stained with annexin V and analyzed by flow cytometry. Quantification was based on three independent experiments. Error bars indicate S.E.M.

It has been reported that besides centrosome over-duplication during S-phase, delay or arrest, unrepaired DNA damage or unfinished DNA replication can lead to the formation of supernumerary centrosomes by centrosome splitting or duplication during a prolonged G2-M phase [26], [31], [35], [37], [41], [43]. GEN1 depletion led to a lower mitotic index but a higher proportion of G2/M cells compared to control cells, suggesting a delay or arrest in the cell cycle phase prior to mitosis (**Fig. 4A**). In accordance with the fact that an increase in the number of centrosomes is only evident in mitotic cells, GEN1 depletion did not result in any apparent S-phase delay (**Fig. 4B–C** **)**. Furthermore, consistent with previous studies prolonged S-phase arrest induced by hydroxyurea (HU) treatment resulted dramatic increase in the level of centrosome amplification (**Fig. S4B**) [35]–[37]. However, simultaneous depletion of GEN1 caused no additional increase in the number of cells with supernumerary centrosomes as compared with the ratio of centrosome amplification between GEN1 deficient and proficient cells without HU treatment (**Fig. S4B**).

Previously, it has been shown that the formation of multipolar spindles can result in mitotic catastrophe, generation of cells with multiple nuclei, aneuploidy and increased apoptosis [43]. In accordance with this, GEN1 depletion resulted in an increase in both the number of cells with multiple nuclei and an increase in apoptosis (**Fig. 4D–E** **)**.

### GEN1 Deficiency Results in Reduced HR of DNA DSBs

**Figure 5:**
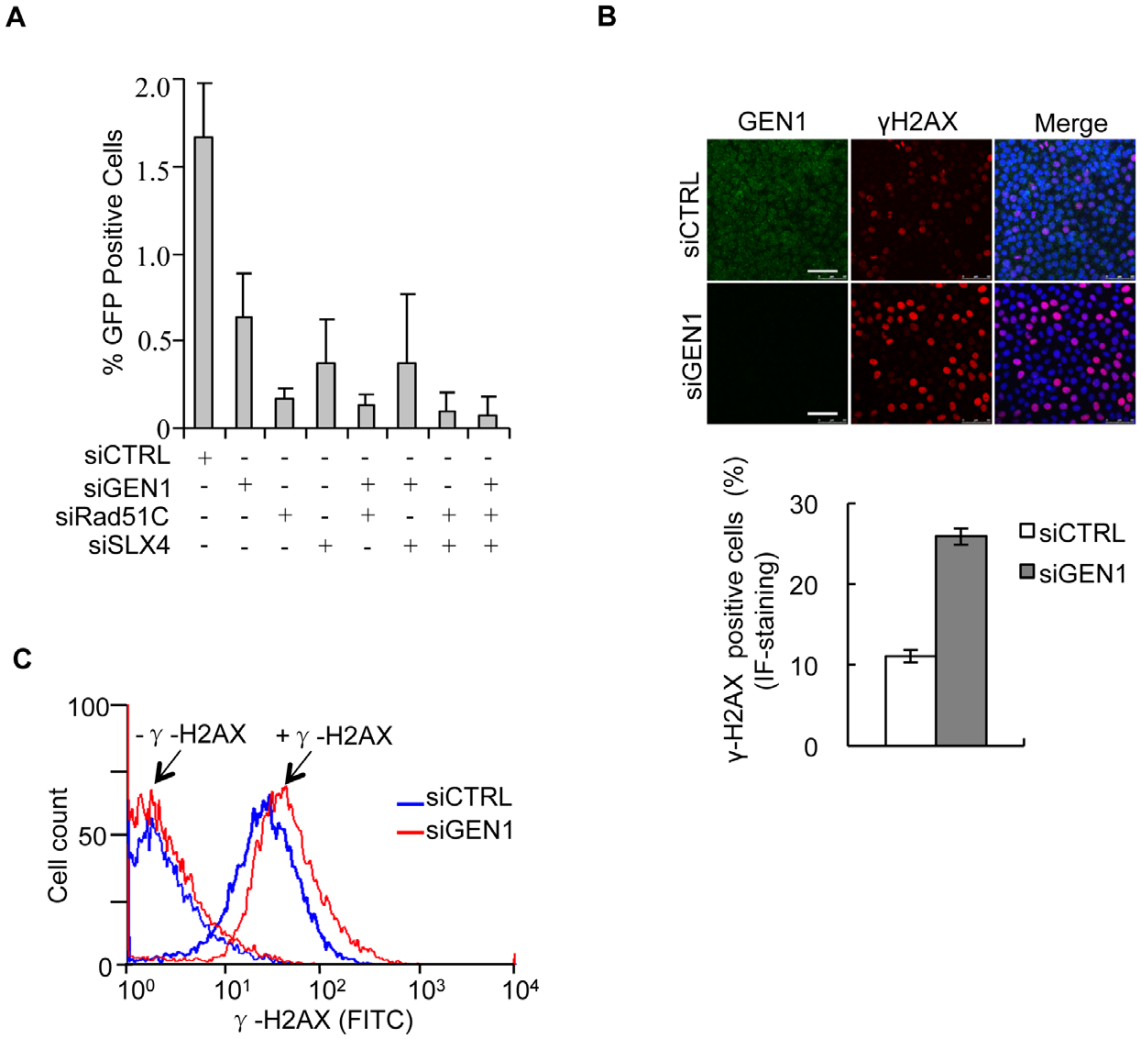
GEN1 is required for HR of DNA DSBs. A. 48 h following siRNA treatment with the indicated siRNAs U20S/DR-GFP cells were transfected with I-SceI plasmid for 48 h. Cells were processed for flow cytometric analysis of GFP, and the extent of HR was scored as the number of GFP-positive cells. Graph represents the mean of three independent experiments. Error bars indicate S.E.M. **B.** HeLa cells treated with CTRL or GEN1 siRNA (48 h) were fixed in ice-cold acetone–methanol (1∶1) and immunostained with GEN1 and γ-H2AX. Scale bar, 100 µm. Graph represents the mean of three independent experiments. (n = 100 cells/condition/experiment). Error bars indicate S.E.M. **C.** HeLa cells were treated with CTRL or GEN1 siRNA for 48 h. After fixation in ice-cold acetone–methanol (1∶1), half the cells were immunostained with γ-H2AX and afterwards all cells were incubated with secondary antibody. The samples were then analyzed by flow cytometry and WinMDI software.

Despite the fact that yeast, *C. elegans*, and human GEN1 have been shown to possess resolvase activity *in vitro* its cellular function in HR of DSBs is still not clear. Even less clear is the potential functional interplay between GEN1 and SLX4/SLX1, the only other vertebrate enzyme complex known to also symmetrically cleave static HJs [15], [16]. To examine the role of GEN1 and its functional relationship with SLX4/SLX1 in HR we employed U2OS cells carrying a DR-GFP substrate [44]. This substrate contains 2 nonfunctional GFP open reading rames, including one GFP coding sequence that is interrupted by a recognition site for the I-SceI endonuclease (SceGFP). Expression of I-SceI leads to formation of a DSB in the I-SceI GFP allele, which can be repaired by HR using a nearby GFP allele lacking N- and C-terminal GFP sequences. This will produce functional GFP, which can be readily detected by flow cytometry [44]. DR-GFP cells treated with control siRNA displayed efficient HR resulting in robust production of GFP-positive cells after I-SceI expression (**Fig. 5A** and **Fig. S4C–D**). However, upon GEN1 depletion HR was reduced by ∼60%, consistent with a role of GEN1 in HR (**Fig. 5A**). Depletion of SLX4, which should be regarded as an SLX1-SLX4 depletion since the stability of SLX1 and SLX4 are interdependent, led to ∼70% reduction in HR (**Fig. 5A**). In accordance with previous findings in *C. elegans* and consistent with a decreased ability to repair DNA DSBs, GEN1 deficiency resulted in an increase in the occurrence of spontaneous DNA damage as measured by the amount of γ-H2AX (**Fig. 5B–C**) [24].

### The Role of GEN1 in Maintaining Centrosome Integrity Appears Separable from its HJ Resolvase Function

**Figure 6:**
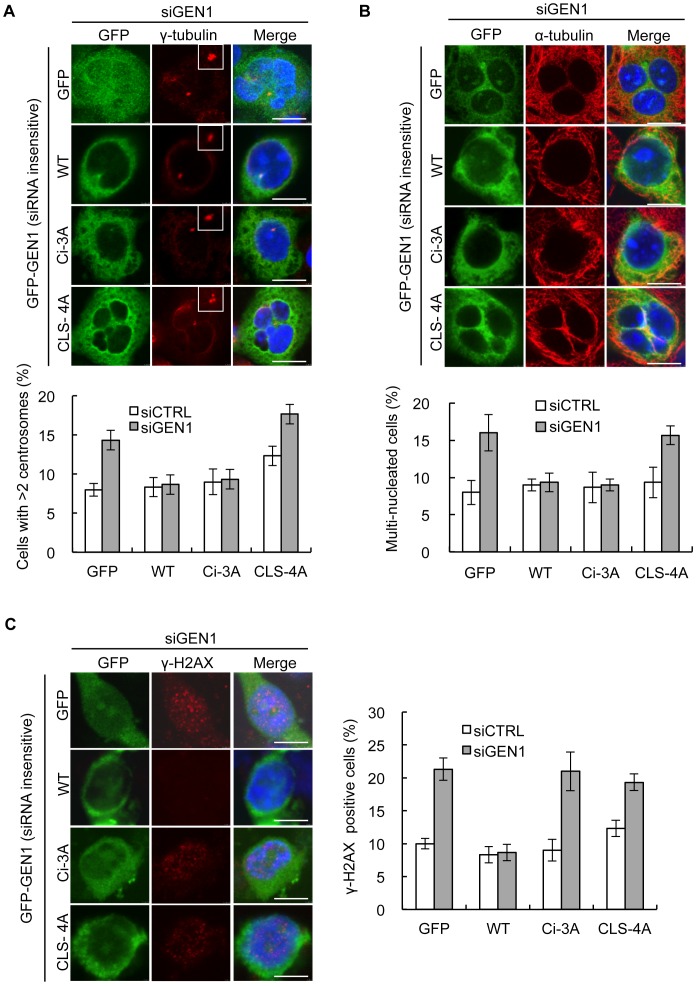
The role of GEN1 in maintaining centrosome integrity appears separable from its HJ resolvase function. A. HeLa cells were simultaneously treated with GEN1 siRNA and the indicated siRNA insensitive GFP-GEN1 constructs for 48 h, methanol fixed and immunostained with the γ-tubulin and DAPI. Quantification was based on three independent experiments. (n = 100 cells/condition/experiment). The white box shows a 6.8 magnification of the centrosome identified by γ-tubulin. Error bars indicate S.E.M. Scale bars, 10 µm. **B.** HeLa cells were treated as in A. but immunostained with α-tubulin and DAPI. Quantification was based on three independent experiments. Error bars indicate S.E.M. (n = 100 cells/condition/experiment). Scale bars, 10 µm. **C.** HeLa cells were treated as in A. but immunostained with γ-H2AX and DAPI. Quantification was based on three independent experiments. Error bars indicate S.E.M. (n = 100 cells/condition/experiment). Scale bars, 10 µm.

In order to investigate if the various phenotypes observed after GEN1 depletion could be assigned to certain functional domains of GEN1, we generated siRNA insensitive GEN1-WT, GEN1-Ci-3A, and GEN1-CLS-4A GEN1 constructs and tested their ability to complement specific defects observed after GEN1 depletion. Strikingly, complementation with WT- or Ci-GEN1 prevented the increase in centrosome numbers and multi-nucleation found in GEN1 depleted cells, whereas expression of the GEN1 mutant GEN1-CLS-4A incapable of localizing to the centrosome failed to do so (**Fig 6A–B**). These findings suggest that the nuclease activity of GEN1 is dispensable for its function at the centrosome. In contrast, only expression of WT but neither of the GEN1 mutants were able prevent the formation spontaneous DNA damage as measured by the percentage of cells showing increased amount of γ-H2AX foci following GEN1 depletion (**Fig. 6C**).

## Discussion

The nuclease activity of GEN1 has be extensively studied and characterized and it has been very convincingly proven that GEN1 can cleave 5′-flaps and four way Holliday junction structures *in vitro* [21]. A delicate phosphorylation dependent cell cycle regulation of GEN1s nuclease activity has also been defined [20]. However, not much focus has been on the cellular functions of GEN1 in a broader context. The present study provides novel insight into the cellular functions of GEN1 and the defects associated with GEN1 deficiency.

As anticipated from the known *in vitro* resolvase activity of GEN1 and consistent with the effect of depleting GEN1 in Bloom’s syndrome cells, we observed a severe defect in HR of DSBs in GEN1 depleted cells (**Fig. 6A**) [21], [22]. Both GEN1 and SLX4 presumably function to resolve dHJs downstream of Rad51 but in an independent fashion. Surprisingly, we did not observe any additive effects when co-depleting GEN1 and SLX4. This could indicate that with regard to HR of DSBs they function in the same pathway. However this would be inconsistent with previous studies and since they display the same specific enzymatic activity towards static HJs *in vitro* we find this unlikely [16], [17], [22]. Rather, we believe that it either reflects the existence of residual protein masking any additional effects or that the loss of the HJ resolvases is compensated for by increased activity of the BLM-TopIII and/or Mus81-Eme1 complex in siGEN1/siSLX4 cells. Alternatively, it may be explained by the existence of a yet not identified protein involved in the resolution/dissolution of HJs, which can act redundantly with GEN1 and SLX1-SLX4. More interestingly, we demonstrate for the first time that GEN1 accumulates on the centrosomes and we identify an N-terminal CLS required and sufficient for the accumulation (**Fig. 1**). Furthermore, we describe novel, very striking and unexpected consequences of defective GEN1 function, namely increased centrosome numbers associated with multi-nuclei formation, DNA damage and increased apoptosis (**Fig. 2A** **and** **Fig. 4D–E**). The fact that ectopic expression of WT- or Ci-GEN1 can complement the centrosomal defect and multi-nucleation associated with GEN1 deficiency, suggests that the role of GEN1 in maintaining centrosome integrity is independent on and separable from its resolvase activity but requires GEN1 to be physically present on the centrosome. In addition, our observations imply that the increase in cells with multiple nuclei is a direct consequence of the increased generation of multipolar spindles in GEN1 depleted cells.

As mentioned previously, various mechanisms for centrosome amplification have already been reported. Multiple centrosomes can arise by centrosome fragmentation or splitting as well as aberrant duplication [26], [31], [35], [37], [41], [43]. We found that the great majority of centrosomes in the multipolar spindles (identified by γ-tubulin) were associated with two Centrin 2 spots, suggesting they had arisen mainly by duplication, rather than by splitting or fragmentation. (**Fig. 3D**). Centrosome amplification can also occur by cell division failure [41], [42], however though we observed an increase in multinucleation (**Fig. 4D**) we did not see an increase in centrosome numbers in interphase cells (**Fig. 3C**). Furthermore, in mitosis the multipolar spindles were often composed of an unequal number of spindle poles, arguing against increased centrosome numbers caused by a cytokinesis failure. The fact that GEN1 depleted cells showed multiple centrosomes only during mitosis but not during interphase, suggests that the centrosomes do not duplicate in the preceding interphase but rather in early mitosis or very late G2-phase. Future examination of GEN1 deficient cells by live cell imaging and electron microscopy will be very interesting, and should help clarify the exact mechanistic underpinnings of the observed centrosome hyper-amplification.

Centrosome amplification during mitosis has previously been observed in cells depleted for Orc2, a component of the origin recognition complex (ORC) [45]. Interestingly, depletion of Orc1 another component of ORC, also results in centrosome amplification, but here amplification is cause by re-duplication of centrioles during S-G2 phase [46]. Together these findings suggest that a link exists to closely coordinate DNA replication with chromosome segregation. The many previous studies showing that centrosome amplification can be caused by DNA damage or deletion of repair proteins defiantly suggest that also DNA damage and repair is closely coordinated with segregation of chromosomes [26], [28], [31], [43]. However, our finding that complementation of GEN1 depleted cells with catalytic inactive GEN1 could prevent supernumerary centrosomes in mitosis (**Fig. 6A**) suggests that GEN1 has a function in controlling centrosome copy number which is independent of its resolvase activity and role in HR.

The catalytic activity of GEN1 is responsible for the function of GEN1 in HR and therefore it is not surprising that complementation with catalytic inactive GEN1 fails to prevent the spontaneous DNA damage caused by GEN1 depletion (**Fig. 6C**). The fact that the CLS-4A mutant can also not prevent spontaneous DNA damage (**Fig. 6C**) might be explained by recent findings by Janssen et al, who demonstrated that chromosome segregation errors frequently leads to damage during cytokinesis resulting in structural chromosome aberrations and DNA breaks [47]. Alternatively, the inability of the CLS-4A mutant to prevent spontaneous DNA damage could also be caused by failure of concentrating this mutant in the vicinity of the mitotic spindle. A recent study showed that the resolvase function of GEN1 is restricted to mitosis, both by direct regulation of the catalytic activity but also by regulating subcellular localization and hence access to joint molecule intermediates [20].

In summary, we have characterized novel functions of human GEN1 and additional phenotypes associated with GEN1 depletion. Most strikingly, we demonstrate that GEN1 is a novel centrosome associated protein that is required for maintaining centrosome integrity. Importantly, it appears that the function of GEN1 in HR is separable from its role in regulating centrosome number, but that they both contribute to maintain genomic stability.

## Materials and Methods

### Cell Culture

Human MRC5 (ATCC, CCL-171), HeLa (ATCC, CCL-2), PANC1 (ATCC, CRL-1469), MCF7 (ATCC, HTB-22) and 293T (ATCC, CRL-11268) cells were grown in RPMI1640 at 37°C, 5% CO2 with 10% fetal bovine serum and 1% penicillin/streptomycin (Invitrogen). Human DR-U2OS cells [48] were grown in Dulbecco’s modified Eagle’s medium with 10% fetal bovine serum. Cells were treated with the following drugs: nocodazole (Sigma; 0.04 ug/ml), ATM inhibitor (Ku55933, KuDos; 10 uM) and hydroxyurea (HU, Sigma; 2,5,10 or 20 mM) at the indicated times.

### Plasmids and RNAi

Total RNA was extracted from 293T cells using Tri-reagent (Sigma). cDNA was synthesized by TaqMan Reverse Transcription Reagents with random primers (Applied Biosystems).The human GEN1(Gene ID: 348654) open reading frame encoding the full-length protein was amplified using the following forward/reverse primers :5′-GAGGCTAGCTGTTTCTGAGGTGGATGTTGAT GTG-3′,5′-AAAACTGCAGATCAAGTGCTTTGGAATCTTA-3′. GEN1 was inserted into plasmid Flag-EGFP-C1B (kindly provided by Jan-Michael Perers, IMP,Vinema) [49] after digest of both plasmid and PCR product with XhoI and PstI. Deletion mutants (1-161aa and 162-908aa) were generated by PCR from FLAG-GFP-GEN1 WT. Point mutations to generate the centrosome accumulation deficient mutant CLS-4A (Y141A/L142A/N143A/V148A) and catalytic inactive Ci-3A (D30A/E134A/E136A/E157A) mutants of GEN1 were introduced using the Quik-change site-directed mutagenesis kit (Stratagene). The following siRNA sequences were used in the study: GEN1 (5′- UAUGCAAACCACUCGGGAAA-3′), (5′-GCGUAAUCUUGGUGGGAAA-3′), (5′-GCCCUAAGAUACAUAUUAA-3′), (5′UCUAAGACCUUUGGCUAUA-3′), Rad51C: (5′-GGC AGUAGAUGUGCAGAUA-3′), (5′-GGGACAUGCUGCUACAAUA-3′), (5′-CUG CUACAAUACGGCUAAU-3′), SLX4: (5′-CCAGCCUGAAAGCCUUAAA-3′), (5′ -CCCACAAGUUCGUGCUUUA-3′), (5′-GCG GAGACUUUGUUGAAAU-3′), ATR: (5′-GCAGAAGCUUAUAGAAAUA-3′), (5′-GGGC AAGGCACAAAUUUCA-3′), (5′-GGGCCGAUUUAUGGAAGAA-3′) and Negative control: (5′-UUCUCCGAACGUGUCACGU-3′). All siRNA duplexes (Dharmacon Research and GenePharma) were used at a final concentration of 20 nM and transfections were performed using Lipofectamine RNAiMAX™ (Invitrogen) according to the manufacturer’s instructions.

### Immunochemical Methods

To get total cell extracts, the pellet containing 1×10^6^ cells were lysed in 100ul RIPA buffer (20 mMTris-HCl, pH 7.4, 20% glycerol, 0.5% NP40, 1 mM MgCl2, 0.5 M NaCl, 1 mM EDTA, 1 mM EGTA, 1 mM DTT, and 1 mM PMSF) and quantified by Bradford reagent. One hundred and twenty micrograms of protein was separated by SDS-PAGE, transferred to PVDF membrane, incubated with primary antibodies in TBST containing 5% non- fat dried milk, and after incubation with HRP–conjugated secondary antibodies, detected by ECL Western Blotting Detection Kit (BD Bioscience). Rabbit polyclonal anti human GEN1 antibodies were raised using GST-GEN1 651–892aa GEN1 (Antibody Research Center, Shanghai Institutes for Biological Sciences, Chinese Academy of Sciences) or peptides spanning amino acids 11-28aa (N-term) or 729–741aa (C-term) of human GEN1 (AbMax Biotechnology Co.Ltd). Briefly, GST tagged GEN1 (651-892aa, pGEX-5x-2-GEN1-C) was expressed in BL21 (CE3)-RIL Competent E. coli, solubilised in Urea buffer and purified using Bio-scale mini profinity GST cartridge columns, as described in the commercial instruction (BioRad BioLogic DuoFlow FPLC protein purification system manual). The purified GST-GEN1 fusion protein was used to immunize rabbits. IgG was purified from the terminal bleeding serum or the control serum using Protein A columns (BioRad). The anti-GST-GEN1 651-892 antibody (1∶2000) was used in all experiments expect for the experiment shown in Figure S2B. Other primary antibodies used in this study included: mouse anti-γ-tubulin antibody (1∶2000; Sigma, T5326), mouse anti-β-tubulin antibody (1∶5000; Sigma, T5293), mouse anti-Rad51C antibody (1∶1000; Abcam, ab2180), rabbit anti-ATR antibody (1∶2000; Abcam, ab2905), mouse anti-ATM antibody (1∶1000; Abcam, ab2618), rabbit anti-phospho Chk1(S317) antibody (1∶1000; Bethyl, A300-163A), rabbit anti-phospho-Chk2(T68) antibody (1∶1000; Cell Signaling, 2661), rabbit anti-Chk1 antibody (1∶2000, Abcam, ab47574) and rabbit anti-Chk2 antibody (1∶2000; Abcam, ab6538).The secondary antibodies used were anti-mouse IgG-HRP antibody (1∶5000; Dakocytomation, p0161) and anti- rabbit IgG-HRP antibody (1∶5000; Dakocytomation, p0448).

### Immunofluorescence

Cells were grown on coverslips, fixed on ice with methanol for 10 min, and permeabilized with 0.1% Triton X-100 and 0.5% NP40 for 10 min on ice, incubated with primary antibody for one hour, washed and incubated with secondary antibody for thirty minutes, and followed were mounted in Vectashield mounting medium with DAPI (Vector Laboratories,Burlingame,CA). The primary antibodies were: rabbit anti-GEN1 antibody (1∶400; homemade), mouse anti-γ-tubulin antibody (1∶500; Sigma, T5326), rabbit anti-CEN2 antibody (1∶400; SantCruze, sc-27793), mouse anti-α-tubulin antibody (1∶1000; Sigma, T9026), mouse anti-(Aurora A) antibody (1∶500; SantCruze, sc-27883), mouse anti-Plk1 antibody (1∶500; SantCruze, sc-17783), mouse anti-CyclinB antibody (1∶200; BD Bioscience, 610220) and rabbit anti-phospho- H2AX (S139) antibody (1∶1000; cell Signaling, 9718).The secondary antibodies were anti-mouse IgG - Cy3 antibody (1∶500; Sigma, c2181) and anti-rabbit IgG – FITC antibody (1∶500; Sigma, f7512).

### Microscopy

Confocal images were acquired on Leica TCS SP5 (Leica) equipped with HCX PL APO 63×1.4 oil CS immersion objective (Leica). Dual color confocal images were acquired with standard settings using laser lines 495 nm and 550 nm for excitation of FITC 495 and Cy3 550 dyes, respectively. Band-pass filters 490–550 nm and 590–630 nm were used to collect the emitted fluorescence signals. Image acquisition and analysis were carried out with SP5 image software.

### Centrosome Isolation

Centrosomes were isolated and fractionated according to the protocol previously described by Meigs et al [50]. In brief, after 90 min incubation with 10 µg/ml nocodazole (sigma) and 5 µg/ml cytochalasin D (sigma), cells were sequentially washed with 1×PBS, 8% sucrose (w/v) in 0.1×PBS, 8% sucrose (w/v) in H2O, 1 mM Tris-HCl pH 8.0 and lysed in lysis buffer (1 mM Tris-HCl (pH 8.0), 0.5% Triton X-100, 0.1% β-mercaptoethanol and proteinase inhibitor mixture (Sigma)). Following centrifugation the supernatant was filtered through a nylon filter (40-µm) and centrifuged on a 20% w/w Ficoll-400 cushion (20% (w/w) Ficoll (MW 400,000, Amersham Biosciences),10 mM PIPES pH 7.2, 1 mM EDTA, 8 mM/0.047% β-mercaptoethanol). The crude centrosomal fraction was collected and transferred to tubes previously padded with a discontinuous sucrose gradient buffer consisting of 5.25 ml of 62.5% (w/v) sucrose solution, 4 ml of 20% (w/v) sucrose solution and centrifuged at 96500 g for 75 min. The sedimented centrosomes were collected in separate fractions containing 100 µl.

### Cell Cycle Analyses

Cells were fixed in 70% ethanol, and resuspended in propidium iodide (PI) buffer (Facsflow (BD), 0.1 mg/ml PI, and 0.02% NaN3). Cell cycle analysis was performed by flow cytometry (FACSCalibur; BD and data analysis was carried out using Modfit LT software (version 3.1; BD).

### G2/M Checkpoint Assay

siRNA-transfected cells were fixed with 70% ethanol overnight, stained with anti-phospho-H3 antibodies (1∶1000, Sigma, ab5176) and propidium iodide(pI), and the mitotic cells were ananlyzed by flow cytometry.

### Apoptosis Assay

Apotosis assay was described previously [51]. 1×10^6^ cells were stained with annexin-7-AAD using the Annexin V PE Apoptosis Detection Kit I (BD Bioscience) and analyzed by flow cytometry. Result was obtained from three independent experiments.

### HR Assay

48 h after siRNA treatment the U2OS derivative cell line harboring an integrated HR reporter construct (DR-GFP) was transfected with a plasmid expressing I-SceI for 48 h. Cells were collected by trypsinization and subjected to flow cytometric analysis of GFP. The extent of I-SceI–induced HR was measured as the number of GFP-positive cells. Non- transfected cells were used a negative control.

### BrdU Incorporation Assay

This assay was described [52].siRNA treated cells were labelled with 10 uM BrdU for 2 hours, return to BrdU-free medium for the indicated times, then fixed with ice-cold 70% ethanol,denatured with 2 M HCl,0.5% Triton X-100, neutralized with 0.1 M Na2B4O7(pH 8.5), stained with FITC conjugated anti-BrdU antibodies (eBiocience) and propidium iodine (PI), and analyzed by flow cytometry.

## Supporting Information

Figure S1 **Generation and characterization of GEN1 antibodies.** **A.** GST-GEN1 (651–892aa) expressed in and purified from BL21 (CE3)-RIL Competent E. coli cells, was analyzed by SDS-PAGE followed by Coomassie blue staining. C: control T: total protein, S: supernatant, E: elution **B.** The specificity of affinity purified GEN1 antibody (immunogen: GST-GEN1) was validated by testing its ability to recognize tagged recombinant GEN1 proteins spanning different parts of GEN1. Purified GST-GEN1-C (651–892aa), His-GEN1-C (651–892aa), and His-GEN1-M (300–600aa) recombinant proteins were analyzed by anti-GEN1, anti-GST or anti-His antibodies. **C.** The specificity of peptide generated C-terminal GEN1 antibody (GEN1 C-term) was tested like in B. **D.** Hela cells were treated with CTRL or GEN1 siRNAs for 48 h and analyzed by immunoblotting with GEN1 antibody (immunogen: GST-GEN1) and β-tubulin.
(TIF)

Figure S2 **Human GEN1 localizes on the centrosome. A.** Cells from the indicated cell lines were methanol fixed and co-immunostained with GEN1 and γ-tubulin antibodies. DNA was visualized by DAPI. Bars: 10 µm. **B.** HeLa cells were methanol fixed and examined by immunefluorescence following co-immunostaining with different GEN1 antibodies (N-term 11-28aa and C-term 729–741aa) and γ-tubulin. Scale bars, 10 µm **C.** HeLa cells were fixed with paraformaldehyde and immunostained with GEN1 antibody and DAPI. Scale bars, 10 µm. **D.** HeLa cells were treated with or without 0.04 ug/ml nocodazole and 14 h later immunostained with GEN1 antibody (anti-GST-GEN1 651–892aa antibody) and α-tubulin antibody, or stained with GEN1 antibody and γ -tubulin antibody. Scale bars, 10 µm. **E.** Sequence alignment of human cyclin E CLS with the putative CLS of GEN1 from various species. The red boxes indicate highly conserved amino acid, four of which were mutated to alanine in the GEN1 CLS-4A mutant Mm, Mus musculus; Rn, Rattus norvegicus; Dm, Drosophila melanogaster; Gg, Gallus gallus; Hs, Homo sapiens; Sc, Saccharomyces cerevisiae.
(TIF)

Figure S3 **GEN1 deficiency results in centrosome amplification. A.** HeLa cells were treated with CTRL or four different GEN1 siRNAs for 48 h, methanol fixed and co-immunostained with the indicated antibodies. **B.** Quantification of mitotic cells with>2 mitotic spindles in the indicated cell lines 48 h after treatment with CTRL or GEN1 siRNA. The histogram shows the percentage of mitotic cells with>2 mitotic spindles from three independent experiments. (n = 300 cells/condition/experiment) Scale bars, 10 µm. **C.** Western blot showing knock down efficiency in HeLa cells 48 h after siRNA transfection. **D.** HeLa cells were treated with CTRL or SLX4 siRNA for 48 h, methanol fixed and immunostained with the indicated antibodies. Graph represents the mean of three independent experiments. (n = 300 cells/condition/experiment). Scale bar, 10 µm.
(TIF)

Figure S4 **GEN1 depletion reduces HDR. A.** MCF7 cells were treated with the indicated siRNAs for 48h, then CPT (5 µM) was added and cells were harvested 1 h later. For ATM inhibition (ATMi), cells were pretreated 1 h with 10 µM ATM inhibitor before CPT incubation. Cells were process for analysis by immunoblotting with the indicated antibodies. **B.** HeLa cells were treated with CTRL or GEN1 siRNA for 48 h and then treated with the indicated doses of hydroxyurea (HU) for another 48 h. After methanol fixation cells were co-immunostained with GEN1 and γ-tubulin antibodies. Histogram represents the mean of three independent experiments. (n = 300 cells/condition/experiment. **C.** 48 h following siRNA treatment with the indicated siRNAs U20S/DR-GFP cells were transfected with I-SceI plasmid for 48 h. Cells were process for analysis by immunoblotting with the indicated antibodies or total RNA was purified and the mRNA levels of SLX4, GEN1, Rad51C were examined by RT-PCR. ARPP served as loading control. **D.** As in C.
(TIF)
