# Supplementary figures and images for: A Novel Role of Human Holliday Junction Resolvase GEN1 in the Maintenance of Centrosome Integrity
Source: PLoS One. 2012 Nov 16;7(11):e49687. doi: 10.1371/journal.pone.0049687 (PMC3500319; doi:10.1371/journal.pone.0049687)

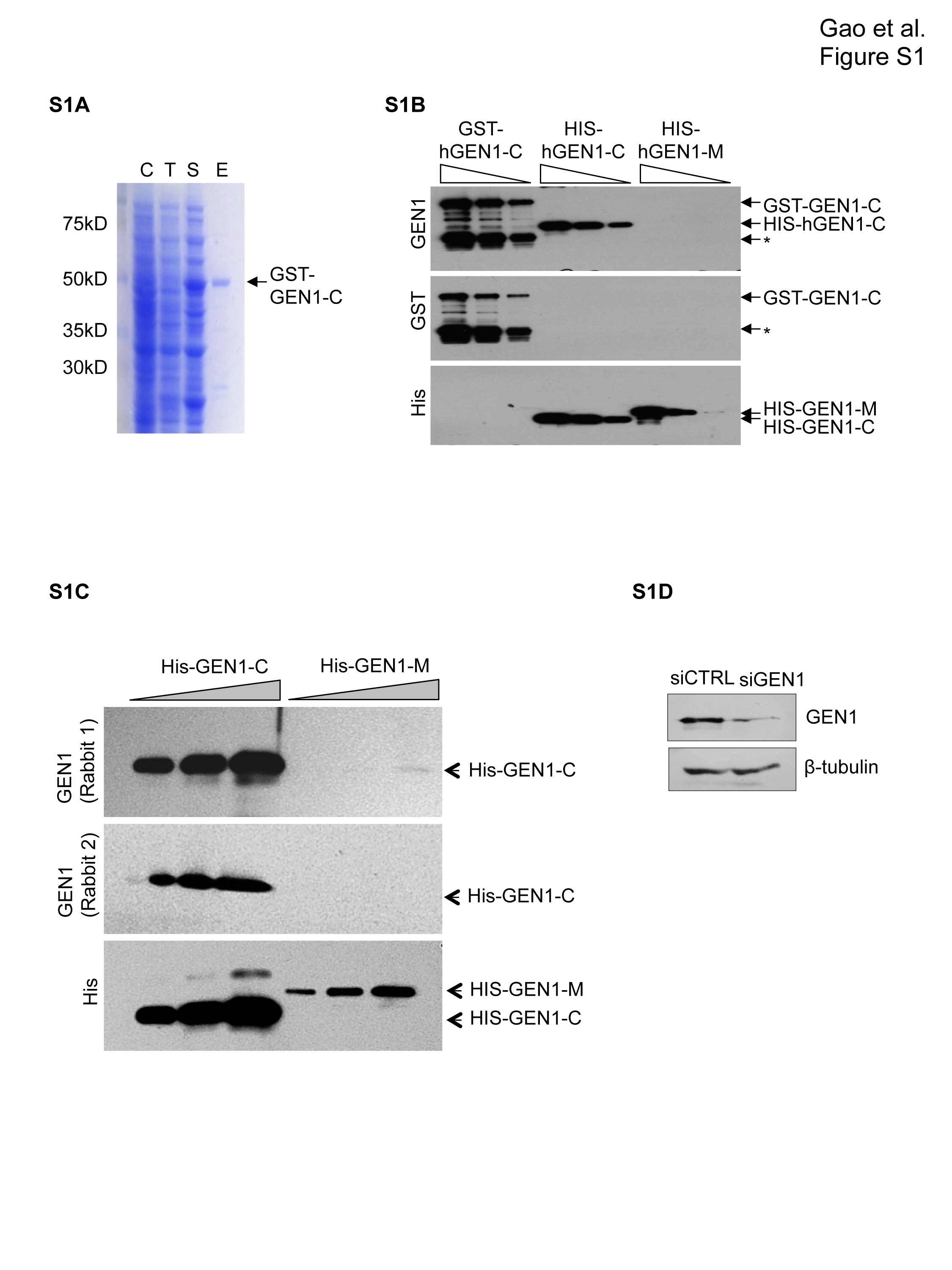

Supplement: Figure S1 — Generation and characterization of GEN1 antibodies. A. GST-GEN1 (651–892aa) expressed in and purified from BL21 (CE3)-RIL Competent E. coli cells, was analyzed by SDS-PAGE followed by Coomassie blue staining. C: control T: total protein, S: supernatant, E: elution B. The specificity of affinity purified GEN1 antibody (immunogen: GST-GEN1) was validated by testing its ability to recognize tagged recombinant GEN1 proteins spanning different parts of GEN1. Purified GST-GEN1-C (651–892aa), His-GEN1-C (651–892aa), and His-GEN1-M (300–600aa) recombinant proteins were analyzed by anti-GEN1, anti-GST or anti-His antibodies. C. The specificity of peptide generated C-terminal GEN1 antibody (GEN1 C-term) was tested like in B. D. Hela cells were treated with CTRL or GEN1 siRNAs for 48 h and analyzed by immunoblotting with GEN1 antibody (immunogen: GST-GEN1) and β-tubulin. (TIF) [file pone.0049687.s001.tif]

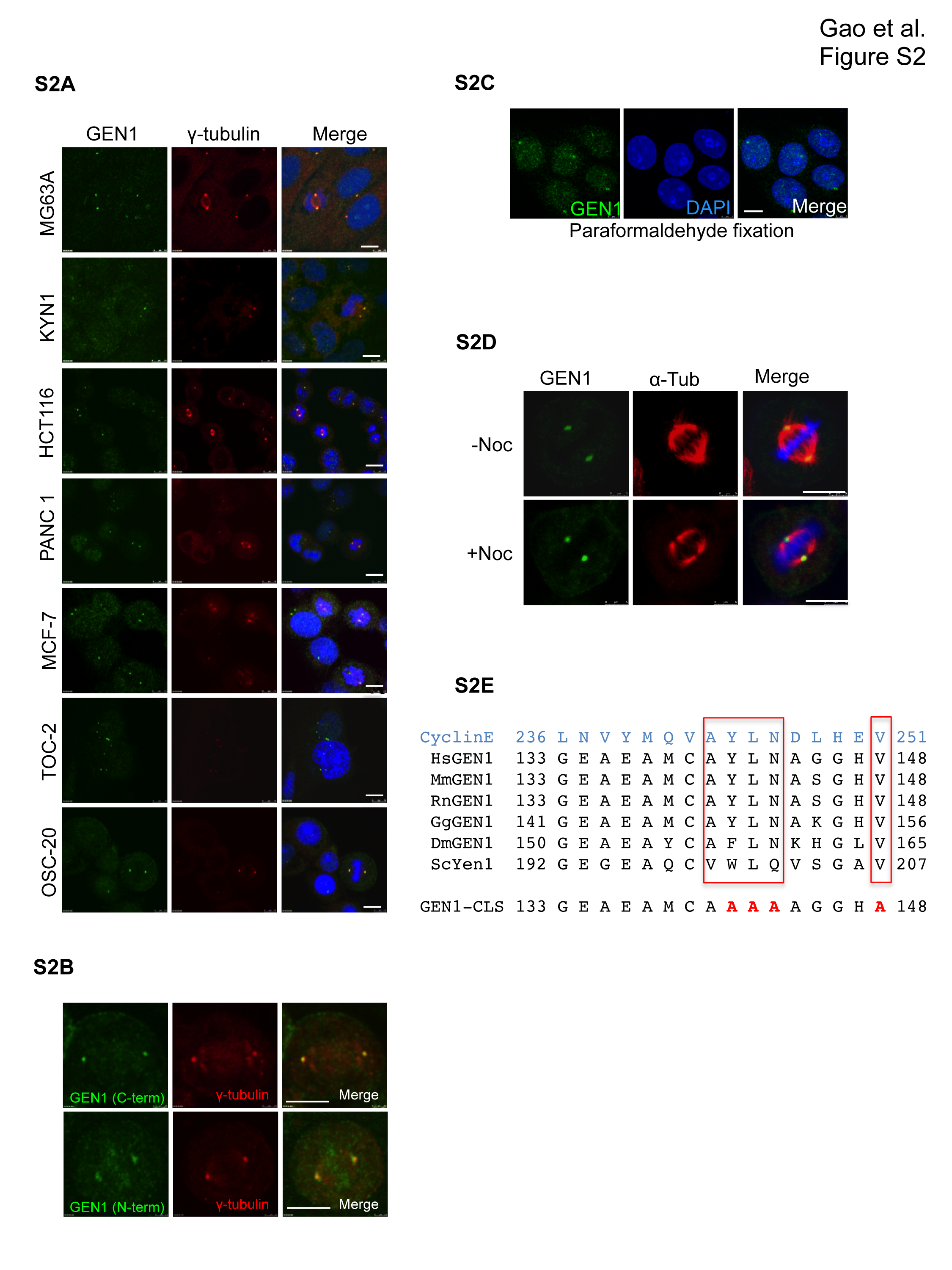

Supplement: Figure S2 — Human GEN1 localizes on the centrosome. A. Cells from the indicated cell lines were methanol fixed and co-immunostained with GEN1 and γ-tubulin antibodies. DNA was visualized by DAPI. Bars: 10 µm. B. HeLa cells were methanol fixed and examined by immunefluorescence following co-immunostaining with different GEN1 antibodies (N-term 11-28aa and C-term 729–741aa) and γ-tubulin. Scale bars, 10 µm C. HeLa cells were fixed with paraformaldehyde and immunostained with GEN1 antibody and DAPI. Scale bars, 10 µm. D. HeLa cells were treated with or without 0.04 ug/ml nocodazole and 14 h later immunostained with GEN1 antibody (anti-GST-GEN1 651–892aa antibody) and α-tubulin antibody, or stained with GEN1 antibody and γ -tubulin antibody. Scale bars, 10 µm. E. Sequence alignment of human cyclin E CLS with the putative CLS of GEN1 from various species. The red boxes indicate highly conserved amino acid, four of which were mutated to alanine in the GEN1 CLS-4A mutant Mm, Mus musculus; Rn, Rattus norvegicus; Dm, Drosophila melanogaster; Gg, Gallus gallus; Hs, Homo sapiens; Sc, Saccharomyces cerevisiae. (TIF) [file pone.0049687.s002.tif]

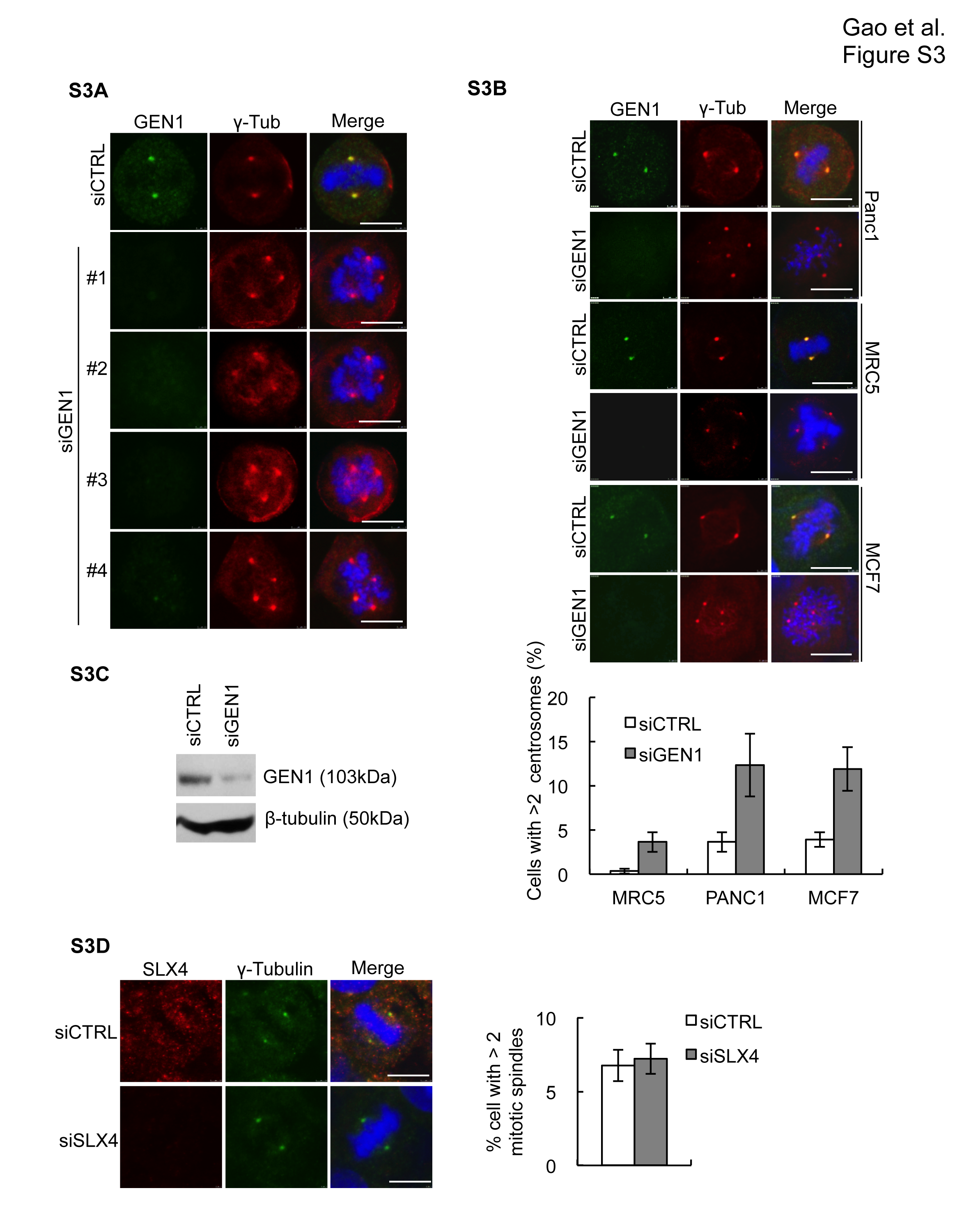

Supplement: Figure S3 — GEN1 deficiency results in centrosome amplification. A. HeLa cells were treated with CTRL or four different GEN1 siRNAs for 48 h, methanol fixed and co-immunostained with the indicated antibodies. B. Quantification of mitotic cells with>2 mitotic spindles in the indicated cell lines 48 h after treatment with CTRL or GEN1 siRNA. The histogram shows the percentage of mitotic cells with>2 mitotic spindles from three independent experiments. (n = 300 cells/condition/experiment) Scale bars, 10 µm. C. Western blot showing knock down efficiency in HeLa cells 48 h after siRNA transfection. D. HeLa cells were treated with CTRL or SLX4 siRNA for 48 h, methanol fixed and immunostained with the indicated antibodies. Graph represents the mean of three independent experiments. (n = 300 cells/condition/experiment). Scale bar, 10 µm. (TIF) [file pone.0049687.s003.tif]

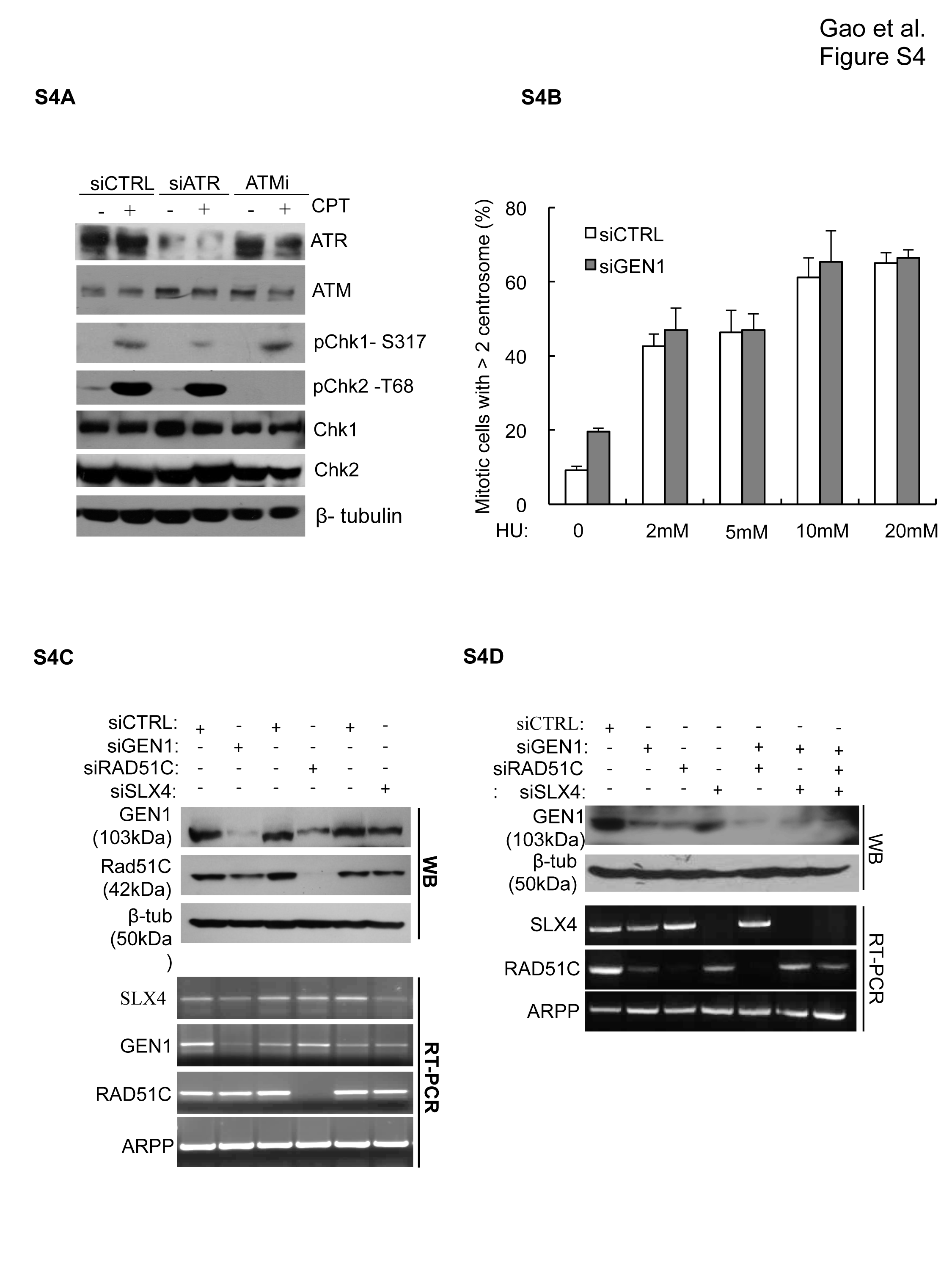

Supplement: Figure S4 — GEN1 depletion reduces HDR. A. MCF7 cells were treated with the indicated siRNAs for 48h, then CPT (5 µM) was added and cells were harvested 1 h later. For ATM inhibition (ATMi), cells were pretreated 1 h with 10 µM ATM inhibitor before CPT incubation. Cells were process for analysis by immunoblotting with the indicated antibodies. B. HeLa cells were treated with CTRL or GEN1 siRNA for 48 h and then treated with the indicated doses of hydroxyurea (HU) for another 48 h. After methanol fixation cells were co-immunostained with GEN1 and γ-tubulin antibodies. Histogram represents the mean of three independent experiments. (n = 300 cells/condition/experiment. C. 48 h following siRNA treatment with the indicated siRNAs U20S/DR-GFP cells were transfected with I-SceI plasmid for 48 h. Cells were process for analysis by immunoblotting with the indicated antibodies or total RNA was purified and the mRNA levels of SLX4, GEN1, Rad51C were examined by RT-PCR. ARPP served as loading control. D. As in C. (TIF) [file pone.0049687.s004.tif]
